# Recovery of expected salary estimated by facial emotion scores against computer-based landscape data

**DOI:** 10.1371/journal.pone.0329757

**Published:** 2026-01-23

**Authors:** Ni Zheng, Haiman Fu

**Affiliations:** 1 Daxing Airport Comprehensive Coordination Office, Beijing, China; 2 Industrial Development Planning Institute of National Forestry and Grassland Administration, Beijing, China; Zhejiang Agriculture and Forestry University: Zhejiang A and F University, CHINA

## Abstract

The perceived recovery of expected salary (RES) matters for work efficacy at a given amount of wage investment. A total of 31 industrial parks (IPs) were randomly chosen from North China. Employees’ facial photos were obtained from social networks and analyzed for happy, sad, and neutral emotion scores. Green spaces were analyzed as surface feature heights and area in 950m-buffer areas at every IP location. Green view index (GVI) was rated using a pre-trained machine-learning model on street view images (SVIs) crawled from the Baidu map. The Simpson diversity index was calculated by recognizing woody plant species in each SVI. RES was estimated as the difference of recruitment wage (mean ± standard deviation, 8625.62 ± 2735.54 CNY M^-1^) minus satisfactory salary (SS) (8153.77 ± 971.28 CNY M^-1^), which was positively impacted by GVI but a negative effect from Simpson plant diversity index. Although the green space area impaired happy score and perception of SS, it enforced a tiny contribution to RES with a negative contribution from the longitude of IPs.

## Introduction

Monthly salaries are strongly related to perceptions of social avoidance, distress, anxiety, and depression among workers [1]. Continuous accumulation of negative moods may result in mental fatigue or even burnout [2]. Sufficient opportunities for interactions with nature can alleviate or reduce the mental stress of employees at the workplace [3]. The theoretical foundations that back this up are derived from the attention recovery theory (ART) and stress reduction theory (SRT). Both theories emphasize the importance of restoring mental health.

A large proportion of the municipal budget is planned to construct, manage, and maintain green spaces to enhance ecosystem services. For city dwellers, urban green spaces can provide immediate opportunities to interact with nature and enjoy mental well-being [4,5]. Evidence has revealed that urban park visitors’ exposure to green spaces can evoke mental restoration and perceived well-being [6–9]. This effect was further identified as a result of the synthesis of effects from the alleviation of the urban heat island effect [10,11], convenient physical accessibility to open spaces [6,12–14], remote location from crowed populations [15–17], comfortable regional meteorological conditions [18–22], and enriched plant species that visitors can experience [23–25]. However, these verifications and identifications were mainly conducted through studies on green spaces in urban parks [14,26,27], where people visit to enjoy leisure time [8,28]. Recently, this effect was identified in people indoors exposed to outdoor green spaces in communities [29]. Taken together, these findings suggest that the landscape attributes of green spaces in the workplace may also lead to positive responses in the emotional perceptions of salary expectations for workers.

Theoretical evidence is insufficient to guide workers’ stress reduction, mainly because the number of participants was limited to a low level of 30–60 [3], moderate levels of 150–220 [3], or rarely over 360 [30]. The subjects in the current study were recruited mainly from office workers (healthcare, consulting, research, technical, managerial, administrative, financial, and professional), the group of whom can rarely represent employees in a wider range of occupations. In a wider causality, the workplace environment can influence job satisfaction [31], which further rules employees’ satisfaction with wages and salaries [32,33] in the social and cultural context regarding individual variation in job performance [32]. However, subjects’ self-reported scores always face queries regarding subjective errors and have a low verification rate [8].

Studies on urban parks have revealed that subtle emotions of visitors can be exposed through facial expressions in the experience of touching nature [34,35]. Compared with self-reported scores on questionnaires, quantifying facial expression scores is a more reliable approach for assessing perceived emotions. Facial photos or selfies can be sources of facial expression scores that can be easily obtained from social networks to pool large datasets [15,36]. Facial expression scores have been successfully used in studies to identify perceived benefits in response to exposure to green spaces in urban parks [15,16,37,38]. Indoor residents were also identified as showing flexible changes in facial expressions in buildings exposed to outdoor green spaces [29]. Together, these results suggest that facial expression scores may also be an available gauge for assessing the perceived well-being of employees at workplaces. Urban park landscape attributes are perceived as variables that can impose visitors’ emotional responses and can be analyzed as geographical metrics of elevation [14], largeness [6,34], surface height [21], location of a city [15,16], proportional ratio in the region [29,34], and plant biodiversity [17,23]. These are also characteristics of landscapes in regions located in industrial parks, and their perceived effects on urban park visitors may also be effective for employees at workplaces. Therefore, it is also applicable to test workers’ perceived emotions about salary expectations regarding their experiences with these landscape metrics at workplaces on a large geographical scale.

A pilot study was conducted, focusing on 31 industrial parks in North China. Employees were considered as subjects whose facial expressions were employed as a novel source of emotional data used to assess salary expectations. The landscape attributes of green spaces in areas around industrial parks were analyzed in terms of geographical metrics, greenness ratio, and plant diversity. Our objectives were to assess the perceived salary expectations of workers regarding landscape metrics in green spaces at workplaces. Our findings will be useful for the management of human resources to control high expectations and limit low salaries about monthly salaries of workers by planning green space landscapes with the aim of controlling the recovery of salary expectations.

## Methodology

### Expeirmental layout and process

**Fig 1:**
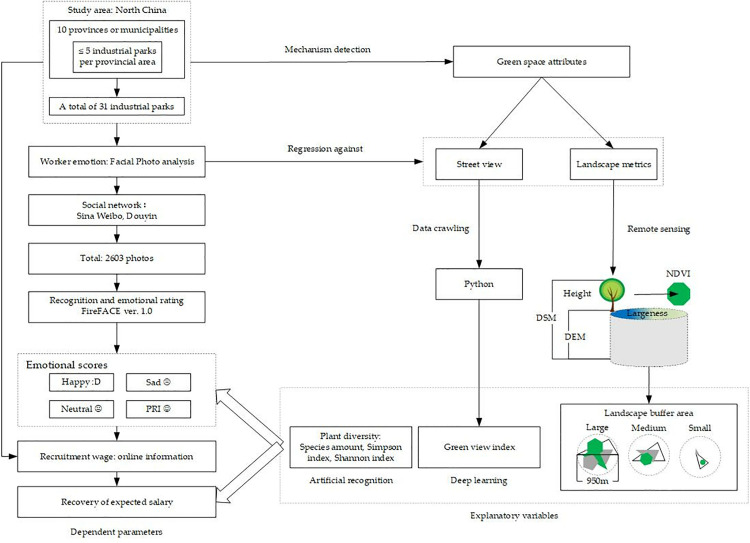
Study design and data analysis process.

As shown in Fig 1, urban industrial parks were chosen as study locations for the provinces and municipalities in North China. Independent variables were employed as greenspace attributes of landscape metrics and street views. The landscape was remotely evaluated using satellite imagery to reveal the area and height of the surface features of green spaces. Street-view images were obtained by data crawling and analyzed for the green view index (GVI) using a deep learning model [39,40]. Street-view images were also artificially analyzed for plant diversity by a professional group [41]. The objective parameter was employed to recover the expected salary (RES), which was estimated by regression against the emotional scores obtained from social networks. Finally, RES and emotional scores were regressed against explanatory variables that would reveal the combined plant diversity and landscape metrics that can elicit emotional perceptions of RES.

### Study sites

The study sites were chosen to be located in the vast areas of North China, namely, the northern lands of the Yellow River. Ten provincial areas were involved in this study, of which 31 industrial parks were chosen as workplaces for data collection. The names and coordinates of the industrial parks are listed in S1 Table in S1 File. The selection of industrial parks depended on the number of workers who displayed facial photos. The details of this are described in the following sections. To extend the spatial distribution to the maximum geographical range, no more than five industrial parks were selected from a province or municipality. This can maximize the latitudinal range of park locations and increase the location-dependent diversity of regional urban planning [42,43].

### Number of subjects

Employed workers were chosen as subjects from January 1, 2022, to December 31, 2023. The invited volunteers delivered their facial photos to the requested online address, with locations exposed at the target industrial parks (IPs) as a source of emotional data. The requirement for consent of the volunteers was waived as they achieved the facts of photo deliveries, which were reviewed and approved by the ethics review committee (20210122UFR). Because of the natural difference of perceived emotions towards the same experience in green spaces among individuals, we are aware of the bias in doses of emotions that human beings would show on their faces in a small number of subjects. Therefore, we designed this study to incorporate at least 50 workers’ faces that can be recognized by software and rated facial expression scores in one industrial park [12,26,27,44]. The maximum number of facial photos was set to be no more than 100 units per park [16,21–23]. That is, if the number of facial photos was not higher than 50 or the number was far higher than 100, the objective industrial park would not be selected. We were also aware that the number of participants determines the precision of assessing perceived emotions against stressful experiences in an industrial park. The number of subjects is important for reducing the impact on bias in the retained data precision. However, an upper limit for the number of photos existed in our data pool.

### Facial photos and emotional analysis

Facial photos of the employees were collected from recruitment delivered by volunteers from the new media of Sina Weibo and Douyin (a Chinese edition of TikTok). In Sina Weibo, photographic style files were directly searched for and downloaded from microblogs exposed at check-in locations in industrial parks [15,34,45] (S1 Table in S1 File). Douyin is a platform where users expose micro-videos and share them with the public or other users on a private friend list. Therefore, micro-videos were searched, downloaded, and grabbed screens for ten shots evenly at ten stops of the living stream. The meaning of these ten shots was taken as the sampling average for a photo at the workplace.

All photos or micro-videos were collected during working times (08:30am–21:00 pm, Monday to Friday with holidays excluded) at locations of objective industrial parks. We assumed that people who exposed their facial photos during this required period were employees of an organization attached to an objective industrial park. We focused on check-in locations at workplaces of objective industrial parks, where selfies were taken mostly by employees and managers during work time. We focused on working days and holidays; weekends were excluded from the chronicle for photo collection. At a spare time, rare people leave the workplaces of objective industrial parks, except for a few security guards. In addition, photos were further screened from the microblogs or original videos according to texts and videos to remove photos uploaded at industrial parks by mistake. The period from 2022 to 2023 was chosen as the time for photo collection in our study to fulfill the basic requirements of the number of photos per industrial park. We could not shorten our sampling period to months or two years [21,46]. Thus, the months in 2022 fell into the COVID pandemic when many companies had to be temporarily stopped, and many on-duty employees wore facial masks; hence, any single dozen months in the past three years cannot be a period long enough for photo collection to a desired number in a single industrial park.

All photographs were initially documented by their photographic locations and rotated to make the nose axis vertical to the basic horizontal line [13]. This operation increases the precision of recognizing facial expressions for each person per photo. A photo with more than one person’s face needs to be cropped to leave only one face, which accounts for approximately 75% of the entire area of a single photo. The sex of the subject in each facial photo was evaluated by a mechanician.

Photos were recognized and analyzed using FireFACE ver 1.0 software to rate happy, sad, and neutral emotions in every facial photo [6,12,16,23,28]. These three basic emotions have been identified and recognized by software, and the matching accuracy of an emotion on a face can meet the requirements of the critical data process [37]. A new variable, the positive response index (PRI), was used to assess the net positive response to an exposure experience, which was calculated as [15]


$$PRI=S_{Happy}-S_{Sad}$$ (1)

where *S*_*Happy*_ is the happy score, and *S*_*Sad*_ is the sad score. Finally, 2603 photos were successfully recognized by FireFACE, and an average of 84 ± 17 photos were used for one industrial park.

### Landscape metrics

Because the objective industrial parks had varied areas, it was meaningless to frame a geographical range for all parks. Therefore, an area buffer was established for all industrial parks that could contain all green spaces as an ecological infrastructure [47,48]. The construction areas of the industrial parks were calculated and compared, which indicated the area of Qingdao Haier S&T Inc. Park was the largest (101.30 ha) and areas in Shenyang Chemical Industrial Park (0.51 ha) and Henan Agr. High-Tech. Park (7.18 ha) was the smallest and medium, respectively. The longest distance between the two ends of Qingdao Haier S&T Inc. The park was measured to be 950m. This fell within a reasonable range of exposure to green space; hence, it was taken as the diameter of the buffer for the round areas of all other industrial parks.

Greenspace areas were remotely evaluated using satellite imagery obtained from the National Aeronautics and NASA Earth Observatory (public domain) [49]. The area of green space was evaluated as the largeness of the normalized difference vegetation index (NDVI), which was calculated as


$$NDVI=\frac{{Band}_{5}-{Band}_{4}}{{Band}_{5}+{Band}_{4}}$$ (2)

where, *Band*_4_ and *Band*_5_ are the surface reflections at the red and near-infrared bands, respectively. Elevation was evaluated using digital elevation model (DEM) data from the GDEM Aster-30m map [50]. The surface feature height was evaluated by subtracting the data from the digital surface model (DSM) [51] and DEM [52]. For a region dominated by green space, the feature height is that of the highest vegetation (usually trees). In the buffered area around one objective industrial park, the DSM-DEM difference can be the height of either a building or a plant [52]. This is the case in our study, in which many objective industrial parks had negative values in the DSM-DEM difference and were excluded from further analysis.

### Green view and plant diversity

Street-view images were used as data sources for analyzing the green view index (GVI) and plant diversity (Fig 1). The GVI parameter is frequently used to quantify the ratio of pixels occupied by plant greenness, accounting for all in an image of a street view [53]. In our study, street-view images were crawled from the Baidu map [54] through an application programming interface (API) [55]. All buffer areas for one industrial park were outlined as a frame (area of 283.39 ha), and the projected space was divided into 400 grids. Each grid has a side length of 84.17m and an area of 0.71 ha. Coordinates were scattered evenly to grids for image crawling locations, and the expected number of images was 100 per industrial park. The green view index was recognized and analyzed by a deep learning model that was pre-trained by a mechanician, with all details disclosed in S1 Fig in S1 File.

A professional group majoring in botanical science was employed to artificially recognize plant species from street-view images. Plant species diversity was assessed using the classical Simpson diversity index (Simpson index) as follows [23]:


$$Simpson\;index=\frac{1}{\sum_{i=1}^{Q}S_{i}^{2}}$$ (3)

where *S*_*i*_ is the ratio of the *i*-species amount to that of all species up to the final *Q*-species. Simpson index values assessed for all street-view images were averaged as the means for the host industrial park.

### Estimate on recovery of expected salary

We define the recovery of expected salary (RES) as the difference between recruitment wage and satisfactory salary (SS). It is common sense that workers would perceive higher satisfaction if they obtained a higher monthly salary; hence, SS has a positive relationship with the dose of exposed happiness, and jointly, a negative relationship with exposed sadness dose. When an employee receives a monthly salary at a level of recruitment wage that is higher than emotionally perceived SS, this salary can be taken as the one meeting the expectation. Conversely, when the paid salary was lower than the SS level, the expectation failed to recover.

Emotional scores usually fail to follow a normal distribution [8,16,28]. SS was estimated by maximum likelihood analysis using the following equation:


$$SS=\sum_{i=1}^{j}\partial_{i}\times Var\left(y_{i}\right)+I_{i}+I^{\prime }$$ (4)

where ∂ is the slope of changes in the estimated SS of *Var*(*y*_*i*_) by the maximum likelihood analysis against an emotional score of *y*_*i*_ for the *i* type of emotion up to a final emotional type *j*; *I*_*i*_ is the intercept of *Var*(*y*_*i*_) from zero (extremely no salary situation) with the perception of the *i* emotion; *and I’* is the intercept of the maximum likelihood analysis model across all types of emotions (*i*…*j*). In our study, *i* was assigned a value of 1 and *j* was assigned a value of 3 because only three basic types of emotions were employed: happiness (*i*=1), sadness (*i*=2), and indifference (*i*=3). The recovery of expected salary (RES) can be calculated as


$$RES=\frac{RW-SS}{RW}\times 100\%$$ (5)

where RW is the recruitment salary collected from recruiting webpages, which equals the amount of salary paid for a worker at a recruited position. RES is a percentage that may be either positive or negative, suggesting recovery of the expected salary or failure of recovery, respectively. When a worker perceives a satisfactory salary to be low, his/her expectation will be high, and RES will be high accordingly. In contrast, when a worker shows positive emotions at a high level of satisfactory salary, his/her salary expectation may be as low as a negative value due to the perception of a large failure to recover the expected salary.

### Statistical analysis

Facial expression score data failed to pass the normality test; hence, they were transformed by ranking to obtain distribution-free [56]. Analysis of variance (ANOVA) was employed to detect the main effects of park variation, season of photographing, gender of subject, and year of data collection on ranked facial expression scores (happy, sad, neutral, and PRI). When significant effects were indicated by ANOVA, emotional values were transformed back to raw data and used to calculate the means. We aimed to unravel the spatiotemporal distributions of facial expression scores in different industrial parks across different seasons using ArcGIS software (Eris-China, Shanghai, China); hence, an interaction was also detected by ANOVA between factors of park variation and season of photography. When the interaction was indicated to be significant, the results were mapped by spatiotemporal patterns; otherwise, the results were only analyzed by main effects. The results were compared using Duncan’s test to cope with the bias between two groups of data with uneven numbers of replicates. Statistical significance was identified at a critical value of 0.05. Facial expression scores were detected for relationships using Spearman correlations. A generalized linear model (GLM) was employed to estimate SS through maximum likelihood analysis. Multivariate linear regression was used to detect ranked data of facial expression scores and raw data of SS against combined topographical (coordinate) and landscape metrics (green space area, DEM (elevation), averaged surface heights in green space or buffered zones), GVI, and plant diversity (species amount and Simpson index).

### Results

#### Spatial distributions of landscape attributes

**Fig 2:**
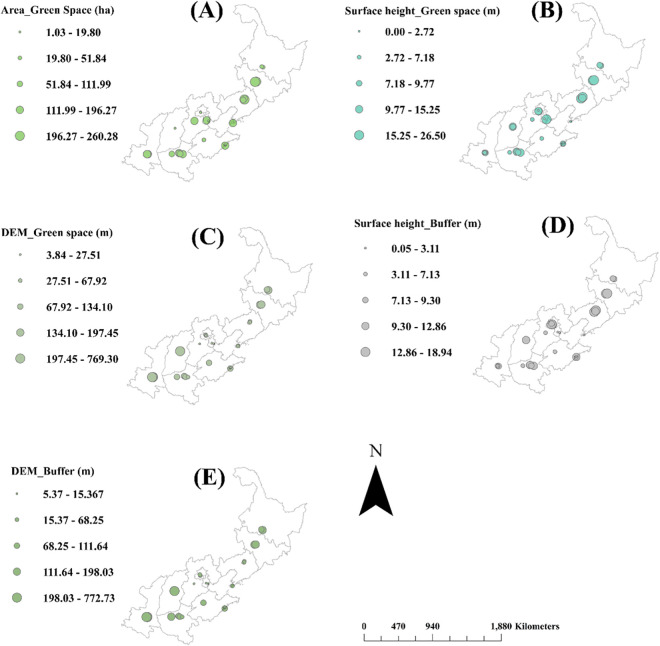
Spatial distributions of landscape metrics in green spaces of buffer areas in industrial parks of North China. Abbreviations: Area_Green Space, green space area; Surface height_Green Space, height of surface features in green space; DEM_Green space, elevation of area in green space; Surface height_Buffer, height of surface features in buffer areas for an industrial park; DEM_Buffer, elevation of buffer area in an industrial park. Mapping data were sourced from NASA Earth Observatory (public domain) (http://earthobservatory.nasa.gov/) which comply with the CC BY 4.0 license.

The green space area in the buffer area was higher in the IPs located in Liaoning and Hebei than in most of the other regions (Fig 2A). The surface feature height in the green space was also higher in the IPs of these two provinces and in Jilin (Fig 2B). However, the area of the buffer zone was higher in the IPs located in the southern provinces of Shanxi and Shaanxi, followed by the IPs of Jilin (Fig 2C). The surface feature height in the buffer zone showed a similar distribution pattern to the height in green spaces, which was higher in Jilin, Liaoning, and Hebei (Fig 2D). Again, the elevation of the buffer zone was higher in Shanxi and Shaanxi than in most of the northern provinces (Fig 2E).

#### Spatial distribution of street view indexes

**Fig 3:**
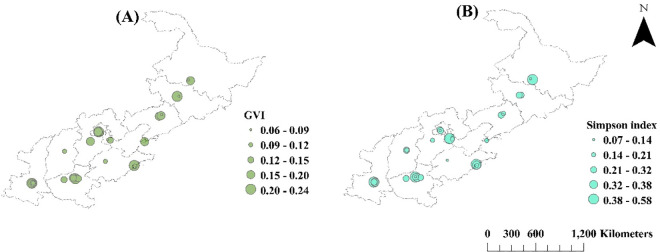
Spatial distributions of green view index (GVI) (A) and Simpson diversity index (Simpson index) (B) in industrial parks of North China. Mapping data were sourced from NASA Earth Observatory (public domain) (http://earthobservatory.nasa.gov/) which comply with the CC BY 4.0 license.

GVI varied between 15% and 20%, with occasionally lower levels (<9%) scattered in IPs in Heilongjiang, Jilin, Liaoning, and Tianjin (Fig 3A). Alternatively, the Simpson index showed high and low levels in IPs along the north-south gradient (Fig 3B). High Simpson index values (>0.38) were found in the IPs in Heilongjiang, Tianjin, Shandong, and Henan, with low values (<0.1) in Heilongjiang, Liaoning, Beijing, Shandong, and Henan.

#### Spatial variations of facial expression scores

**Fig 4:**
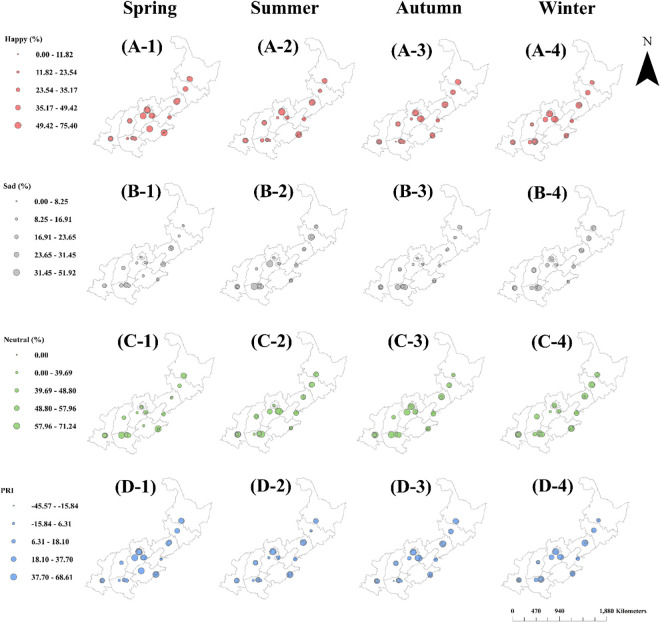
Spatiotemporal distributions of workers’ facial expressions scores in industrial parks at regions of North China across four seasons. (A-1) to (A-4), happy score distributions; (B-1) to (B-4), sad score distributions; (C-1) to (C-4), neutral score distributions; (D-1) to (D-4), PRI score distributions. Mapping data were sourced from NASA Earth Observatory (public domain) (http://earthobservatory.nasa.gov/) which comply with the CC BY 4.0 license.

ANOVA indicated interactive effects between park location variation and investigation season on facial expression scores (S2 Table in S1 File). Therefore, the spatial distributions of the facial expression scores were drawn across the four seasons, and the results are shown in Fig 4. High levels of happiness scores (>49%) were mostly distributed in IPs in the agglomerated regions of Liaoning, Beijing, and Tianjin in most seasons (Fig 4 A-1 to A-4). In contrast, sad scores were lower (<8%) in the IPs of these regions, with high levels (>23%) distributed in the IPs at Hebei and Henan in Summer (Fig 4 B-2) and winter (Fig 4 B-4). Neutral scores were higher (>48%) in Henan in most seasons of the year (Fig 4 C-1 to C-4), and in the agglomerated regions of Beijing, Tianjin, and Hebei in Summer (Fig 4C-2) and autumn (Fig 4C-3). High PRI scores (>37%) were found in IPs in Shandong and Beijing during most seasons (Fig 4D-1 to D-4). High PRI scores also existed in the agglomerated regions of Beijing, Tianjin, and Hebei in Spring (Fig 4D-1).

**Fig 5:**
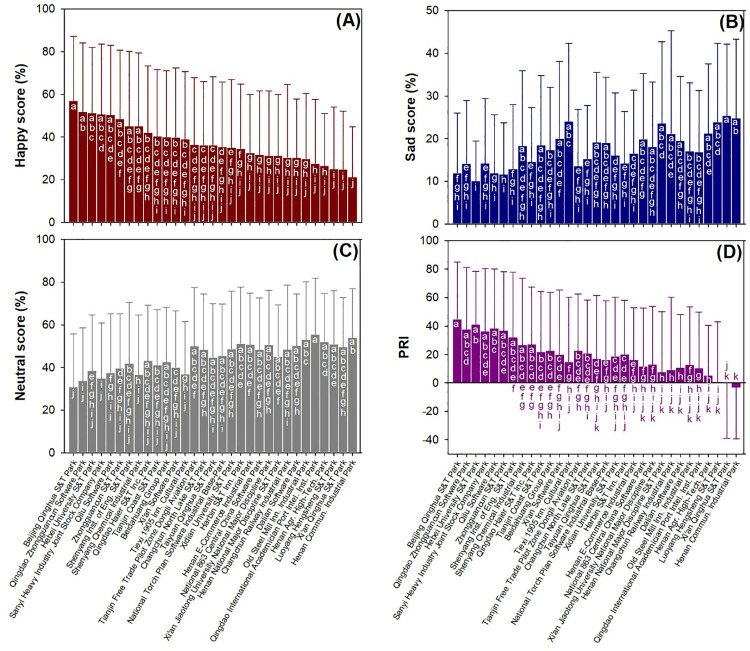
Facial expression scores for workers among industrial parks for happy (A), sad (B), neutral (C), and PRI (D) scores. Different lower-case letters indicate significant difference using Duncan test at 0.05 level. Data were transformed by ranking in difference detection, but they are transformed back to raw values and used for calculating averages for industrial parks.

Employees showed higher happiness scores in Beijing Qinghua S&T Park than in most other IPs, and employees in two IPs at Henan showed the lowest levels of happiness scores (Fig 5A). Sad scores showed a general ascending trend in descending order among IPs for happy scores with occasional fluctuations (Fig 5B). Hebei University S&T Park showed lower sad scores than most of the other IPs. The Tiexi 1905 Innovative Cultural Park showed a higher average sad score than the Tianjin Free Trade Pilot Zone Dongli Aviation Park and Changchun North Lake S&T Park. Neutral scores showed a generally similar trend among IPs to that of sad scores (Fig 5C). In contrast, PRI scores showed a descending trend among IPs compared to sad and neutral scores (Fig 5D). Xi’an Qinghua S&T Park and Henan Communication Industrial Park showed PRI scores as low as negative values.

Neutral scores showed significant differences among seasons (S2 Table in S1 File), and scores in summer and autumn were higher than those in spring (S2 Fig in S1 File). Gender also had a significant effect on facial expression (S2 Table in S1 File). Female employees showed higher happy scores than males (S3 Fig A in S1 File); in contrast, female employees’ sad and neutral scores were lower than those of males (S3 Fig B, C in S1 File). Again, female employees showed higher PRI scores than male employees (S3 Fig. D in S1 File).

Both happy and PRI scores had negative relationships with sad and neutral scores, whose coefficient of determination (*R*^2^) ranged from 0.52 0.80 (S4 Fig in S1 File). Sad scores showed a positive relationship with neutral scores, with *an R*^2^ of 0.14. Happy score also had a positive relationship with the PRI score, with a high *R*^2^ of 0.96.

#### Estimate of RES and spatial variation

According to the maximum likelihood analysis, happy, sad, and neutral scores were input into the model for regressing the SS. Only records of happy (*y*_*happy*_) and sad scores (*y*_*sad*_) were regressed to have significant estimates of RES, which can be calculated using the following equation:


$$SS=122.85\times y_{happy}-3905.01-12.93\times y_{sad}+8000.00$$ (6)

**Fig 6:**
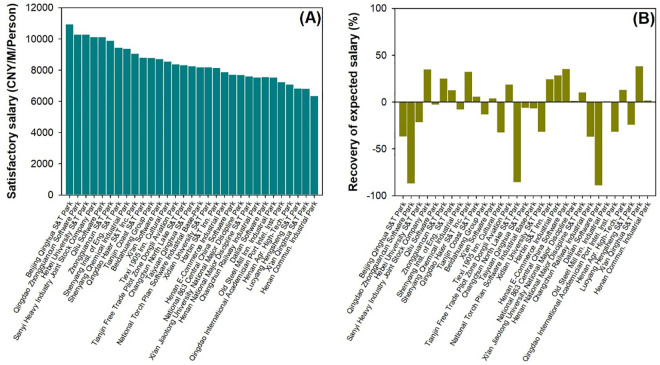
Variations of satisfactory salary (A) and recovery of expected salary (B) among industrial parks.

**Fig 7:**
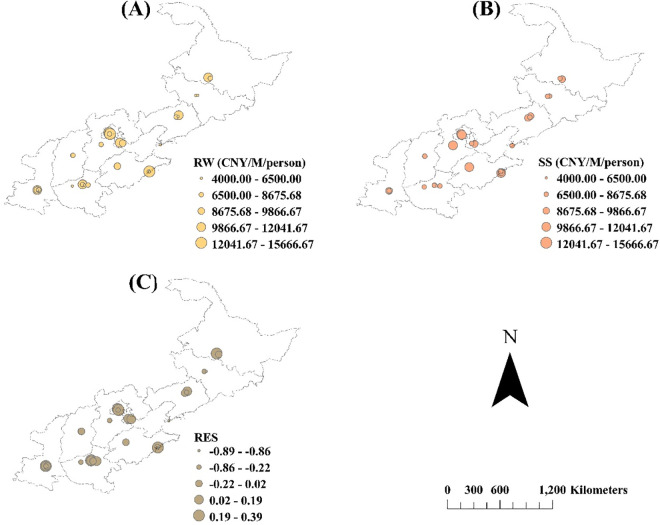
Spatial distributions of recruitment wage (RW) (A), satisfactory salary (B), and recovery of expected salary (RES) (C) in industrial parks located at regions of North China. Mapping data were sourced from NASA Earth Observatory (public domain) (http://earthobservatory.nasa.gov/) which comply with the CC BY 4.0 license.

The results for the SS and RES are shown in Fig 6, and their distributions are shown in Fig 7. High RWs (>12000 CNY) were mainly provided by IPs in Beijing and Shandong, whereas parts of the IPs in Heilongjiang, Liaoning, Tianjin, Henan, and Shaanxi provided medium levels of RW (8600–9800 CNY) (Fig 7 A). Generally, SS showed a similar descending trend among IPs in an order like that for happy scores (Fig 4 A; Fig 6 A), resulting from the positive contribution from happy scores and the negative contribution from sad scores. High SS values were observed in the IPs of Beijing, Hebei, and Shandong (Fig 7 B). However, RES showed a high fluctuation following the order of IPs (Fig 6B). The IPs of Qingdao Zhongguancun Software Park, Tianjin Free Trade Pilot Zone Dongli Aviation Park, and Dalian Software Park all showed extremely low RESs, with negative values close to −90%. IPs of the Sanyi Heavy Industry Joint Stock Company Park, Qingdao Haier S&T, Inc. Park, National 863 Central China Software Park, and Xi’an Qinghua S&T Park showed high RESs of approximately 40% (Fig 6 B). Most provinces had positive RES values, except for Jilin, Hebei, Shandong, and Henan, where RESs values were as low as <−22% (Fig 7C). However, the IPs in Heilongjiang, Beijing, Tianjin, Shandong, Henan, and Shaanxi had RES at high levels of 2–39%.

#### Regressions of facial expression scores and SS

The combined greenspace area and Simpson index jointly contributed negatively to the ranked happiness scores (Table 1). The Simpson index also made a negative contribution to the ranked neutral scores. Sad scores were positively correlated with GVI. Again, the green space area made a negative contribution to ranked PRI scores with another positive contribution from IPs’ latitudes of the IPs.

**Table 1:** Coefficients in multivariate linear regressions of ranked facial expression scores for happy, sad, and neutral emotions for people living in industrial parks in Northern China.

Dependent	MLR ^a^ coefficient	Intercept	GreenA ^b^	GVI ^c^	Simpson ^d^	Latitude	Longitude
**Happy** ^e^	PE ^f^	27.91	−3.97 × 10^−6^	–	−25.43	–	–
	*SE* ^g^	3.90	1.82 × 10^−6^	–	10.51	–	–
	*F* ^h^	51.19	4.76	–	5.85	–	–
	*P* ^i^	<0.0001	0.0378	–	0.0223	–	–
**Sad** ^e^	PE	4.05	–	75.92	–	–	–
	*SE*	5.01	–	30.34	–	–	–
	*F*	0.65	–	6.26	–	–	–
	*P*	0.4255	–	0.0182	–	–	–
**Neutral** ^e^	PE	8.58	–	–	24.92	–	–
	*SE*	3.70	–	–	11.29	–	–
	*F*	5.39	–	–	4.87	–	–
	*P*	0.0274	–	–	0.0353	–	–
**PRI** ^e^	PE	−14.36	−4.10 × 10^−6^	–	–	0.91	–
	*SE*	15.59	1.82 × 10^−6^	–	–	0.40	–
	*F*	0.85	5.06	–	–	5.25	–
	*P*	0.3649	0.0326	–	–	0.0297	–
**SS** ^j^	PE	10085.00	−5.20 × 10^−4^	–	−3553.17	–	–
	*SE*	490.84	2.29 × 10^−4^	–	1322.67	–	–
	*F*	422.11	5.15	–	7.22	–	–
	*P*	<0.0001	0.0312	–	0.0120	–	–
**RES** ^k^	PE	2.73	1.70 × 10^−7^	–	–	–	−0.02
	*SE*	1.19	7.07 × 10^−8^	–	–	–	0.01
	*F*	5.30	4.92	–	–	–	4.92
	*P*	0.0290	0.0349	–	–	–	0.0349

^a^ MLR, multivariate linear regression.

^b^ GreenA, green space area.

^c^ GVI, green view index.

^d^ Simpson, Simpson biodiversity index.

^e^ Happy, Sad, Neutral, PRI ranked values used for these four types of emotional scores.

^f^ PE, MLR parameter estimate.

g *SE*, standard error.

h *F*, *F* values for the MLR PE.

i *P*, *P* value for MLR PE.

^j^ SS, salary satisfaction index.

^k^ REW, recovery of expected salary.

The Simpson index and green space area also had negative effects on SS (Table 1). In contrast, the green space area made a positive contribution to RES, with a negative contribution from the longitudes of IPs.

## Discussion

### Estimate of RES based on RW and SS

Our RW fell in a range between 4000.00 CNY M^-1^ and 15666.67 CNY M^-1^ with an average (± standard deviation) of 8625.62 ± 2735.54 CNY M^-1^ (coefficient of variation (CV): 0.32). This wage level is higher than the average level (5436.42 CNY M^-1^) by 58.66% in private organizations in cities and towns of China by 2022 [57]. Positions recruited by corporations in IPs in this study were mainly looking for knowledge workers (engineering, senior manager, data analyzer, etc.) working in the office. Corporates in our IPs were mainly from software, high-tech, e-commerce, communication, aviation, and heavy manufacturing industries, which have higher capacities to provide higher salaries than those in other industries such as agriculture, the environment, water conservation, hotels, and hospitality [57]. Corporates in the domains of our IPs all engage in e-commercial retailing, which makes a significant contribution to increasing regional gross domestic product (GDP) [58]. However, office knowledge workers were also characterized as a group of people who easily suffered mental stress and perceived low well-being. Hence, they needed a higher salary satisfaction as a reward to respond to their stressful experiences at work, according to Resource Drain Theory [59].

According to our estimation, the highest SS (10287.53 CNY M^-1^) was lower than that in RW by 34.33%, but the lowest SS (6378.77 CNY M^-1^) was higher than that in RW by 59.47%, with an average of SS to be 8153.77 ± 971.28 CNY M^-1^ (CV: 0.12). These results suggest that an emotion-based estimate of salary can be satisfied at an upper limit than at recruitment; however, employees are all satisfied with a higher salary. It can also be explained that employees who received high salaries did not look as happy as those who received low salaries. High mental stress results in high salaries for positions in an IP with dual challenges and profits. Although our SS was estimated based on regression using WR against multiple emotional scores, the data variation was lower in SS than in WR. In equation (6), the involvement of the sad score with a negative coefficient also accounted for lower satisfaction with high salaries because more negative emotions were exposed by high-income employees.

We found that the IPs in Beijing and Qingdao were higher in RW, SS, and RES. Although RESs in Harbin, Shenyang, and Henan were also ranked higher than 19%, these resulted from low SS in these regions, but not high RW. Beijing Zhongguancun S&T Park and Qingdao Haier S&T Inc. Park had higher capacities to provide salaries with a higher recovery of expectations from employees who were also satisfied with their paid salaries.

### Driving forces of green space structure on facial expressions and SS

Surprisingly, our results demonstrated the negative effects of greenspace area and plant diversity on positive emotions. Compared to the parameter estimate of the green space area (−3.97 × 10^-6^ ± 1.82 × 10^-6^), that for the Simpson index was much lower (−25.43 ± 10.51). This suggests that plant diversity made a heavier driving force to induce depression on happy scores than the green space area. Plant diversity in urban parks has been found to be a major trigger for visitors to perceive improved mood states and subjective well-being [17,23]. In a survey across five European cities, people were reported to prefer higher plant species richness in streetscapes, parks, and wastelands, which agreed that higher plant species richness allows for more livable cities. The Simpson index made a positive contribution to neutral scores that shared a comparable level of absolute value (24.92 ± 11.29) as for happy scores. Together, these results demonstrate that diverse plants at workplaces were perceived as triggers of indifferent sentiments rather than happy emotions. This contradicts the findings in urban parks [23], where people spend time on a visit to perceive leisure, but people spend time in the workplace to earn household income at the cost of mental stress accumulation; hence, employees mostly do not care about plant diversity.

However, high GVI caused a response of perceptible sadness shown on the faces of workers, suggesting that workers do not like plant greenness existing in their workplaces too much. These findings failed to agree with those on GVI perceived by visitors to urban parks [13]. A higher ratio of green views along a path will make visitors perceive higher doses of nature in urban parks, which improves mood states by upregulating unintended attention and reducing mental stress [60,61]. At workplaces, however, people preferred good access to green spaces established with larger open spaces, but not a high rate of green spaces that occupied views of visible accesses. Workers may be more satisfied with the openness towards green spaces rather than seeing crowded green spaces in an IP.

It was interesting to find that latitude had a positive contribution to PRI, which was the difference between the happy and sad scores. This suggests that workers in the northern IPs look happier than those in the southern IPs. Because the green space area had a jointly negative contribution to PRI, we can speculate that northern IPs had larger areas of green space but lower levels of GVI than IPs in the south to promote happy scores and decrease sad scores, respectively. These speculations can be confirmed by the gradient of green space areas and GVIs in the IPs along the north-south gradient. Lower air pollution and lower heat island effects can also account for the higher PRI in northern IPs [22,46]. The generally low perceptions about pressures from installment payments for house loans may also be a reason why northern workers show higher PRI.

Again, the green space area and Simpson index showed dual contributions to SS, which resulted from their depressing forces on the expressions of happy scores, as SS was estimated to be positively changed with happy scores. In Equation (6), SS was calculated with a happy score with a coefficient of 122.85, but with a sad score only with a coefficient of 12.93. The low contribution of the sad score to SS excluded GVI from the regression due to its low effect. In contrast, RES was regressed with a positive contribution from the greenspace area because RES was the difference between RW and SS. Longitude also had a jointly negative contribution to RES, which resulted from a dual effect that eastern IPs had smaller green space areas that depressed perceptions of high RES. These results suggest that a larger green space area can benefit the expectations of employees about their satisfaction, which are mainly distributed in IPs in the western region.

## Conclusions

In the provinces and municipalities of North China, employees’ positive emotions were mostly exposed to industrial parks in Beijing, Tianjin, and Qingdao, while negative and neutral emotions were mostly shown in industrial parks in the south during most seasons of the year. Recovery of expected salary can be calculated as the difference of recruitment wage (8625.62 ± 2735.54 CNY M^-1^) minus satisfied salary (8153.77 ± 971.28 CNY M^-1^), which was averaged to be −3.3 ± 28.29%, suggesting most employees perceived that their salaries failed to meet their expectations. The green plant view should be controlled at workplaces to prevent workers from perceiving sad emotions. Diverse plants were not necessary, as they could induce more indifferent sentiments on employees’ faces than smiles. Although large areas of green space can impair employees’ positive emotions, they can increase their perceived recovery of expected salaries. Finally, large areas of green space should be encouraged in the IPs of North China.

## Supporting information

S1 File **Table S1.** Summary of industrial parks (*n* = 31) with details of location, name, area, coordinate, and number of subjects who exposed facial expressions in North China. **Table S2.** Analysis of variance (ANOVA) of industrial park location (Park, P), season of investigation (Season, S), and their interaction (P × S), as well as gender of subjects and year of facial photo collections on facial expression scores for happy, sad, and neutral emotions of subjects who exposed facial photos in industrial parks of North China. **Figure S1.** Process of street view image analysis for quantifying green view index through a pre-trained deep learning model. **Figure S2.** Dynamic changes of facial expression scores for happy (A), sad (B), neutral (C) emotions and PRI scores (D) across four seasons. Error bars mark standard errors. Different lower-case letters show significant difference according to Duncan test at 0.05 level. **Figure S3.** Whisky-box plots of facial expression scores for happy (A), sad (B), neutral (C) emotions and PRI scores (D) between female and male employees in IPs at regions of North China. Hollow dots mark 5% (top) and 95% quantiles (bottom) and the dash line indicates mean values. Different letters indicate significant difference between genders according to Duncan test at 0.05 level. **Figure S4.** Nomograph revealing relationships between facial expression scores for happy, sad, neutral emotions and PRI scores. Full lines in red color indicate positive relationships and those in blue indicate negative relationships.
(RAR)
